# Validation Parameters of Patient-Generated Data for Digitally Recorded Allergic Rhinitis Symptom and Medication Scores in the @IT.2020 Project: Exploratory Study

**DOI:** 10.2196/31491

**Published:** 2022-06-03

**Authors:** Stephanie Dramburg, Serena Perna, Marco Di Fraia, Salvatore Tripodi, Stefania Arasi, Sveva Castelli, Danilo Villalta, Francesca Buzzulini, Ifigenia Sfika, Valeria Villella, Ekaterina Potapova, Maria Antonia Brighetti, Alessandro Travaglini, Pierluigi Verardo, Simone Pelosi, Paolo Maria Matricardi

**Affiliations:** 1 Department of Pediatric Respiratory Medicine, Immunology and Critical Care Medicine Charité-Universitätsmedizin Berlin Corporate Member of Freie Universität Berlin and Humboldt-Universität zu Berlin Berlin Germany; 2 Pediatric Allergology Unit Sandro Pertini Hospital Rome Italy; 3 Allergology Service Policlinico Casilino Rome Italy; 4 Translational Research in Pediatric Specialities Area, Division of Allergy Bambino Gesù Children's Hospital Scientific Institute for Research, Hospitalization and Healthcare Rome Italy; 5 Department of Immunology and Allergy Santa Maria degli Angeli Hospital Pordenone Italy; 6 Department of Biology University of Rome Tor Vergata Rome Italy; 7 Center of Aerobiology Regional Agency for the Protection of the Environment Pordenone Italy; 8 TPS Production Rome Italy

**Keywords:** allergic rhinitis, symptom scores, patient-generated data, patient-reported outcomes, mHealth, mobile health, health applications, allergies, allergy monitor, digital health, medication scores

## Abstract

**Background:**

Mobile health technologies enable allergists to monitor disease trends by collecting daily patient-reported outcomes of allergic rhinitis. To this end, patients with allergies are usually required to enter their symptoms and medication repetitively over long time periods, which may present a risk to data completeness and quality in the case of insufficient effort reporting. Completeness of patient’s recording is easily measured. In contrast, the intrinsic quality and accuracy of the data entered by the patients are more elusive.

**Objective:**

The aim of this study was to explore the association of adherence to digital symptom recording with a predefined set of parameters of the patient-generated symptom and medication scores and to identify parameters that may serve as proxy measure of the quality and reliability of the information recorded by the patient.

**Methods:**

The @IT.2020 project investigates the diagnostic synergy of mobile health and molecular allergology in patients with seasonal allergic rhinitis. In its pilot phase, 101 children with seasonal allergic rhinitis were recruited in Rome and instructed to record their symptoms, medication intake, and general conditions daily via a mobile app (AllergyMonitor) during the relevant pollen season. We measured adherence to daily recording as the percentage of days with data recording in the observation period. We examined the patient’s trajectories of 3 disease indices (Rhinoconjunctivitis Total Symptom Score [RTSS], Combined Symptom and Medication Score [CSMS], and Visual Analogue Scale [VAS]) as putative proxies of data quality with the following 4 parameters: (1) intravariation index, (2) percentage of zero values, (3) coefficient of variation, and (4) percentage of changes in trend. Lastly, we examined the relationship between adherence to recording and each of the 4 proxy measures.

**Results:**

Adherence to recording ranged from 20% (11/56) to 100% (56/56), with 64.4% (65/101) and 35.6% (36/101) of the patients’ values above (highly adherent patients) or below (low adherent patients) the threshold of 80%, respectively. The percentage of zero values, the coefficient of variation, and the intravariation index did not significantly change with the adherence to recording. By contrast, the proportion of changes in trend was significantly higher among highly adherent patients, independently from the analyzed score (RTSS, CSMS, and VAS).

**Conclusions:**

The percentage of changes in the trend of RTSS, CSMS, and VAS is a valuable candidate to validate the quality and accuracy of the data recorded by patients with allergic rhinitis during the pollen season. The performance of this parameter must be further investigated in real-life conditions before it can be recommended for routine use in apps and electronic diaries devoted to the management of patients with allergic rhinitis.

## Introduction

Digital and mobile health technologies are creating new perspectives in many areas of research and medical care. One important aspect in both fields is the ability to easily collect patient-generated data via smartphone apps and connected devices such as wearables, diagnostic tools, or environmental sensors [1-5]. Although the use of patient-reported outcomes has become popular over the last decade [6-9], it is not sure how accurately the collected data represent the patient’s state, as recording is done without supervision [10,11]. In particular, daily reporting over a longer time period may be perceived as challenging and cause a certain degree of reporting fatigue. The risk of potentially lower quality owing to disengaged survey respondents has been described with different terms, most recently in the field of psychology as *“insufficient effort reporting”* [11,12]. However, consensus on methodologies assessing the quantity and quality of entered data is still missing. Proposed methodologies include the (1) response pattern approach [13], (2) response time approach [14], (3) infrequency approach [15], (4) inconsistency approach [16], and (5) the number of unanswered questions. Most of these methodologies, however, refer to single points of data collection and extended questionnaires, which make their application in a setting with daily data recording via smartphone apps difficult to impossible. As digital methods of data collection via openly available mobile apps usually generate very large data sets, new challenges for the analysis and interpretation apply, such as the lack of standard measures [17]. The importance of unified approaches to data recording has recently been underlined in the context of patient adherence, and computational solution approaches were published to support uniform data formats [18]. A representative example for the daily acquisition of patient-generated data are mobile apps related to seasonal allergic rhinitis [19,20]. A variety of apps has been published for patients with pollen allergies, providing exposure forecasts, individualized symptom prediction, symptom and medication diaries, and in some cases, the possibility of exchanging recorded data with the attending physician [3,10,21-23]. Although several studies have shown the potential of mobile technologies for research purposes and clinical disease management, only few address the topic of data quality and validation [24,25]. The purpose of this study is to retrospectively investigate 4 putative validation criteria to assess the quality of data longitudinally collected by patients with seasonal allergic rhinitis and defined as follows: (1) intravariation index, (2) percentage of zero values, (3) coefficient of variation, and (4) percentage of changes in trends. To this end, we have taken advantage of the patient-generated symptom and medication data set, which has been acquired via a mobile app in a cohort of patients with seasonal allergic rhinitis in the context of the @IT.2020 pilot project [26,27].

## Methods

### Study Design

The @IT.2020 pilot project is an observational clinical study on the impact of component-resolved diagnosis and digital symptom recording on the diagnosis of pollen allergy. In the context of this project, 101 patients experiencing seasonal allergic rhinitis were recruited in the Sandro Pertini Hospital in Rome. The detailed study protocol has been published previously [26,27]. Briefly, recruited patients underwent a medical examination first (T0), including skin prick testing, blood sampling, and clinical questionnaires. At the end of the visits, patients were instructed on the use of the AllergyMonitor (TPS Software Production) mobile app to monitor their symptoms of the eyes, nose, and lungs, as well as medication intake and the impact of allergy symptoms on their daily activities during an individual study period. After the monitoring period, all patients underwent a second medical examination (T1), including a repetition of the initial clinical questionnaires focused on the past pollen season.

### Ethics Approval

The study design and procedures were approved by the local ethics committee “Comitato Etico Indipendente Lazio 2” (study 10-16, Protocol 9871—01/02/2016).

### AllergyMonitor App

AllergyMonitor is a CE-certified smartphone app designed for the daily reporting of allergic symptoms of the eyes, nose, and lungs. Further, the impact of allergic symptoms on daily activities and sleep as well as the medication intake were recorded. In order to facilitate the correct recording of medication intake, the study doctor registered the patients’ individual medication via the back end of the app, and the patient’s tailored drug name and administration schedule appeared in the app’s front end.

### Symptom and Medication Scores

**Figure 1:**
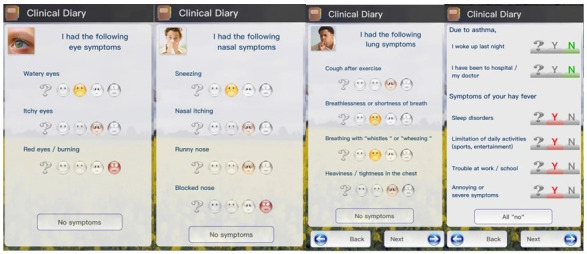
Screenshots of the AllergyMonitor app, indicating the emoticons used to assess symptom severity.

The following symptom and symptom medication scores were used in this study: Rhinoconjunctivitis Total Symptom Score (RTSS, 0-18 points) [28], Combined Symptom and Medication Score (CSMS, 0-6 points) [29], and Visual Analogue Scale (VAS, 0-10 points) [30]. RTSS and CSMS were calculated automatically by the app for every reporting day on the basis of 4 questions on nasal symptoms (sneezing, rhinorrhea, nasal pruritus, nasal congestion), 2 questions on ocular symptoms (itchy eyes, watery eyes), and 3 questions on medication intake (antihistaminic drugs, local steroids, systemic steroids). The severity of each of the symptoms was also measured by the patient using 4 different emoticons (Figure 1), each one representing a distinct severity grade (no symptoms, mild, moderate, or severe). Overall, severity was also measured using VAS in response to the question “How do you feel in relation to your allergic symptoms today?”

### Adherence to Electronic Diary Recording

Retrospective reporting of symptoms for missed days was only possible within 48 hours. After 2 days of missed reporting, an automated reminder appeared on the patient’s phone. After 3 days of missed recording, the patient received an individual email or phone call from the study center to ensure that no technical problems had occurred. Adherence was measured as the percentage of days with completed electronic diary recording in the monitoring period [26]. Patients with adherence to recording above or below the arbitrary threshold of 80% were defined as high or low adherence, respectively.

### Validation Parameters

Data retrospectively obtained were summarized as numbers (n) and frequencies (%) if they were categorical and as mean (SD) or median (IQR) if quantitative. The prevalence of atopic sensitization to airborne allergens was evaluated for every patient via skin prick test results (cutoff for positivity: a wheal size of ≥3 mm). For every pollen period considered, adherence values were calculated for each patient. Four tentative parameters for validation of the data quality were calculated as follows.

#### Intravariation Index

For each i^th^ subject (*i = 1,,,,n*), (1) the percentage of the number of variations between 2 consecutive days was calculated, 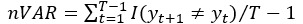, where *t*= 1,,,,,,, T is the indicator of time point considered, y is the value of the symptom medication score considered, and I is a binary variable, which is 1 when the condition in the brackets is verified, 0 otherwise; (2) the individual variation range was calculated (rVAR = (max(y1, …, yT) – min(y1, …, yT))/S, where S represents the unit increase for each symptom medication score, that is, S=1 for VAS and RTSS and S=0.167 for CSMS); and (3) the average of all individual intravariation index values was calculated, 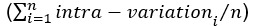, for each symptom score or symptom medication score.

#### Percentage of Zero Values

Average of individual percentages of zero values (100*number of zero compiled values/actual compiled days)

#### Coefficient of Variation

Average of individual coefficient of variation (100*SD/mean)

#### Percentage of Changes in Trend

Average of individual percentages of changes in severity trends (worsening=plus; stability=zero; improvement=minus) between 2 consecutive values of symptom medication score

### Statistical Analysis

Spearman rank correlation coefficient was used to investigate the relationship between individual averages for each symptom medication score considered. The nonparametric Mann-Whitney *U* test was applied to compare the average values of quality indexes between 2 groups of subjects divided by their adherence of recording (<80% vs ≥80%). *P*<.05 was considered statistically significant. Statistical analyses were performed with R Core Team (2018) version 3.5.2 (The R Project for Statistical Computing).

## Results

### Study Population and Pollen Season

This analysis consisted of 101 children (mean age 13.7 [SD 2.8] years) meeting the inclusion criteria for the @IT.2020 pilot study. Male gender was slightly more frequent (63/101, 62.4%), and the population was characterized by predominantly persistent allergic rhinitis symptoms by Allergic Rhinitis and its Impact on Asthma criteria, as assessed by retrospective questionnaire during T0. In addition to persistence, the severity of symptoms for 39.6% (40/101) of the patients was classified as moderate-to-severe (Table 1). The rate of patients with moderate-to-severe persistent symptoms increased to 70.3% (64/101) at the final study visit when being asked the same questionnaire concerning the past pollen season. The most frequent allergic comorbidities were oral allergy syndrome (32/101, 31.7%), atopic dermatitis (28/101, 27.7%), and allergic asthma (28/101, 27.7%). Most patients were sensitized to grass pollen, with 97% (98/101) having a positive skin prick test to Timothy grass and 90.1% (91/101) reacting to Bermuda grass (Table S1 of Multimedia Appendix 1). Grass pollen concentrations ranged from 0 to 199 grains/m^3^ air. Season criteria of the European Academy of Allergy and Clinical Immunology [31] were adapted to the local setting, and the dates of whole grass pollen season from April 13 to July 28 as well as the peak grass pollen season between May 4 and June 28, 2016 are reported in this study (Figure S1 of Multimedia Appendix 1).

**Table 1:** Characteristics of the study population (N=101).

Characteristics			Value
Males, n (%)			63 (62.4)
Age (years), mean (SD)			13.7 (2.8)
**Allergic rhinitis**			
	Age at onset (years), median (IQR)		6 (4-8)
	**Allergic rhinitis and its impact on asthma classification at first medical examination, n (%)**		
		Mild intermittent	19 (18.8)
		Mild persistent	31 (30.7)
		Moderate/severe intermittent	11 (10.9)
		Moderate/severe persistent	40 (39.6)
	**Allergic rhinitis and its impact on asthma classification at second medical examination, n (%)**		
		Mild intermittent	6 (6.6)
		Mild persistent	17 (18.7)
		Moderate/severe intermittent	4 (4.4)
		Moderate/severe persistent	64 (70.3)
**Other allergic comorbidities, n (%)**			
	Allergic asthma		28 (27.7)
	Oral Allergy Syndrome		32 (31.7)
	Urticaria/angioedema		19 (19.2)
	Atopic dermatitis		28 (27.7)
	Gastrointestinal disorders		4 (4)
	Anaphylaxis episode		10 (10.1)
	Other		5 (5.1)

### Adherence to Recording

During the grass pollen season (May 4 to June 28, 2016), 4003 single reports were collected, equaling an average adherence to recording of 70.8% (4003/5654). Over the period of 56 days, the individual number of filled questionnaires ranged from 11 (20%) to 56 (100%); 65 of the 101 patients (64.4%) were highly adherent to data collection. A delayed reporting start or an anticipated end [27] was observed, with 9 patients starting the monitoring 3 days or more after the start of the prescribed period and 12 patients ending the reporting ≥3 days before the prescribed end. Figure S2 of Multimedia Appendix 1 shows that 53 patients had a prescribed monitoring starting before the grass pollen season.

### Interrelation Among RTSS, CSMS, and VAS

**Figure 2:**
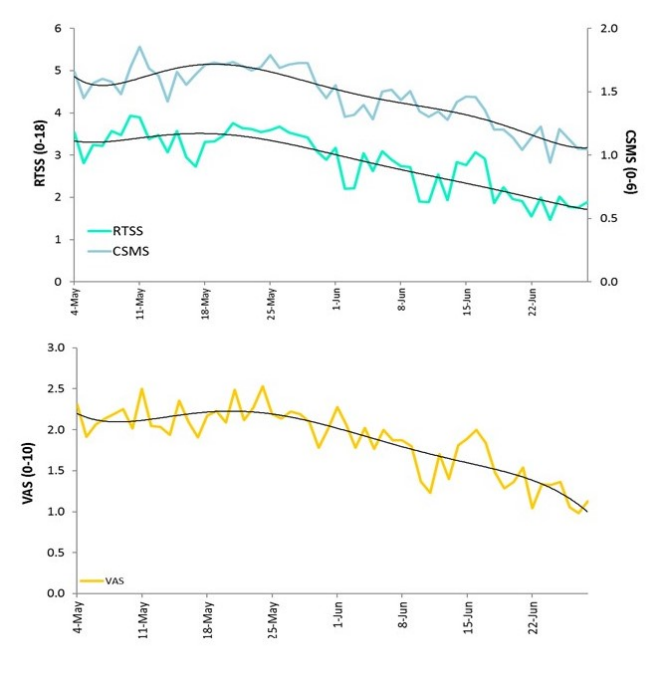
Average population values of Rhinoconjunctivitis Total Symptom Score (0-18 points), Combined Symptom and Medication Score (0-6 points) (both top panel), and Visual Analogue Scale (0-10 points) on impact of allergic symptoms on daily life (bottom panel) over time. CSMS: Combined Symptom and Medication Score; RTSS: Rhinoconjunctivitis Total Symptom Score; VAS: Visual Analogue Scale.

**Figure 3:**
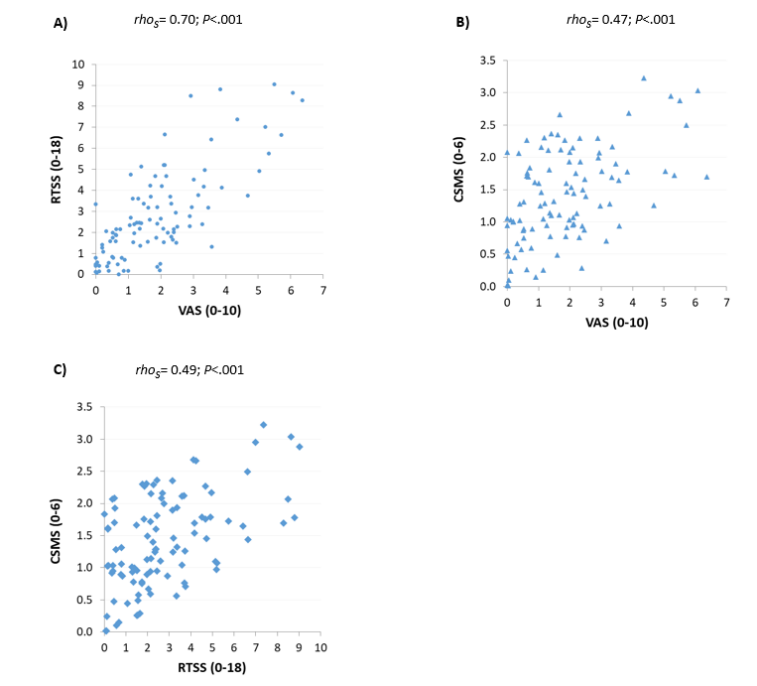
Correlation between individual averages of (A) Rhinoconjunctivitis Total Symptom Score versus Visual Analogue Scale, (B) Combined Symptom and Medication Score versus Visual Analogue Scale, and (C) Combined Symptom and Medication Score versus Rhinoconjunctivitis Total Symptom Score. CSMS: Combined Symptom and Medication Score; RTSS: Rhinoconjunctivitis Total Symptom Score; VAS: Visual Analogue Scale.

The RTSS and CSMS correlate well at a population level over time (Figures 2 and 3). Although these 2 scores are calculated based on identical symptom questions with and without the integration of symptomatic medication, the VAS depicted in the bottom panel takes information from a separate question, filled with the same frequency and showing a similar trend over time. At the individual level, the average VAS score correlated well with both—the average symptom score (RTSS) and the average symptom-medication score (CSMS) (Figure 3A and B).

### Quality Indices in RTSS, VAS, and CSMS

**Figure 4:**
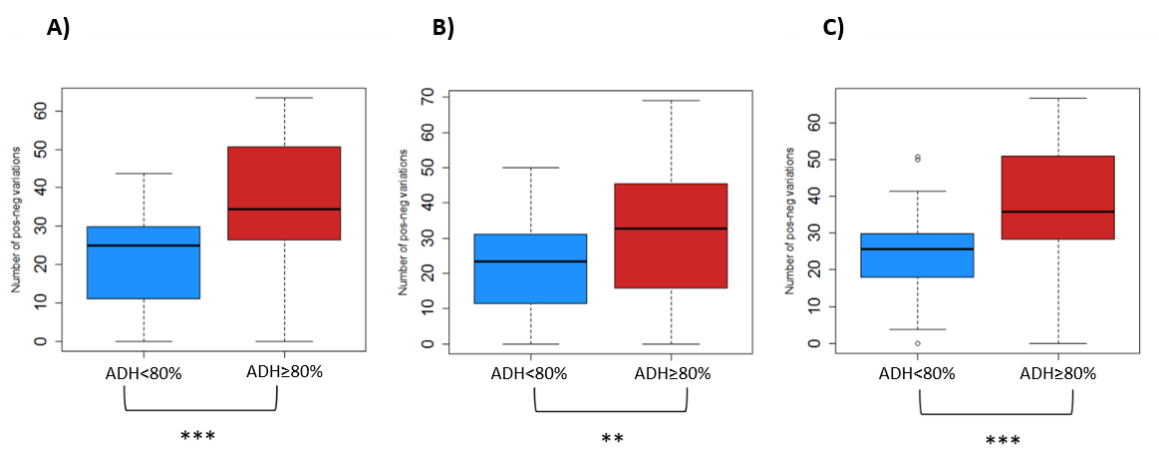
Changes in trends (positive/negative changes) of recorded (A) Rhinoconjunctivitis Total Symptom Score, (B) Visual Analogue Scale, and (C) Combined Symptom and Medication Score values among patients with adherence to recording of <80% versus ≥80%. ADH: adherence. **P<.01; ***P<.001.

In order to assess the quality of recorded data, 4 different parameters were investigated within each of the 3 scores (RTSS, VAS, CSMS) (Table 2), and average values between highly and poorly adherent patients were compared (Table 3, Figure 4).

The highest diversity of data was observed in the VAS and the RTSS, as expressed by the intravariation index and the coefficient of variation. However, the CSMS was more homogeneous over time. As expected, the percentage of zero values was the lowest in the CSMS, whose average values were almost half of those observed in the RTSS and VAS. By contrast, the percentage of changes in the trend was quite similar and high for all the 3 scores, with values oscillating around 30% (Table 2).

**Table 2:** Quality of symptom and symptom-medication scores.

Quality index	Rhinoconjunctivitis Total Symptom Score, mean (95% CI)	Visual Analogue Scale, mean (95% CI)	Combined Symptom and Medication Score, mean (95% CI)
Intravariation index^a^	5.1 (4.5-5.6)	6.1 (5.5-6.7)	2.9 (2.6-3.2)
% of zero values^b^	38.9 (33.3-44.5)	43.3 (37.2-49.3)	20.4 (16.0-24.8)
Coefficient of variation^c^	134.8 (116.5-153.0)	138.1 (115.8-160.5)	86.3 (72.3-100.3)
% of changes in trends^d^	31.3 (28.2-34.4)	28.9 (25.6-32.3)	32.7 (29.6-35.7)

^a^Average of intravariation index by subjects; for each ith subject, (1) the percentage of the number of variations between 2 consecutive days is calculated, 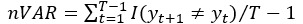, where *t*= 1,,,,,,, T is the indicator of time point considered, y is the value of the symptom medication score considered, and I is a binary variable, which is 1 when the condition in the brackets is verified, 0 otherwise; (2) the individual variation range has been calculated (rVAR = (max(y_1_, …,y_T_) – min(y1, …, y_T_)) + 1/S, where S represents the unit increase for each symptom medication score, that is, S=1 for Visual Analogue Scale and Rhinoconjunctivitis Total Symptom Score and S=0.167 for Combined Symptom and Medication Score); and (3) the average of all individual intravariation index values was calculated, 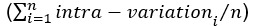, for each symptom score or symptom medication score.

^b^100*number of zero compiled values/actual compiled days.

^c^Average of individual coefficient of variation (100*SD/mean).

^d^Number of changes in trends (plus/minus/stable) within ith differences between 2 consecutive values of symptom medication score.

**Table 3:** Association between adherence and quality indexes.

	Rhinoconjunctivitis Total Symptom Score				Visual Analogue Scale				Combined Symptom and Medication Score		
	Adh^a^<80% (n=36), median (IQR)	Adh≥80% (n=65), median (IQR)	*P* value^b^	Adh<80% (n=36), median (IQR)		Adh≥80% (n=65), median (IQR)	*P* value	Adh<80% (n=36), median (IQR)		Adh≥80% (n=65), median (IQR)	*P* value
Intravariation index^c^	4 (3-6)	5 (4-6)	.08	5 (4-8)		7 (4-8)	.18	3 (2-3)		3 (2-4)	.32
% of zero values^d^	27 (12-72)	38 (17-55)	.66	22 (16-60)		43 (22-65)	.31	13 (0-31)		16 (4-28)	.61
Coefficient of variation^e^	105 (79-197)	99 (79-131)	.76	84 (60-115)		115 (76-158)	.06	77 (53-95)		74 (48-101)	.67
% of changes in trend^f^	25 (11-30)	34 (26-51)	<.001	23 (11-31)		33 (16-45)	.004	26 (18-30)		36 (28-51)	<.001

^a^Adh: adherence.

^b^Mann-Whitney *U* test was used to compare means among the 2 groups.

^c^Average of intravariation index by subjects; for each ith subject, (1) the number of variations between 2 consecutive days has been calculated, 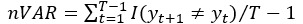, where *t*= 1,,,,,,, T is the indicator of the time point considered, y is the value of the symptom medication score considered, and I is a dummy variable, which is 1 when the condition in the brackets is verified, 0 otherwise; (2) the individual variation range has been calculated (rVAR = (max(y_1_, …,y_T_) – min(y1, …, y_T_)) + 1/S, where S represents the unit increase for each symptom medication score, that is, S=1 for Visual Analogue Scale and Rhinoconjunctivitis Total Symptom Score and S=0.167 for Combined Symptom and Medication Score); and (3) the average of all individual intravariation index values was calculated, 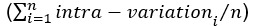, for each symptom score or symptom medication score.

^d^100*number of zero compiled values/actual compiled days.

^e^Average of individual coefficient of variation (100*SD/mean).

^f^Number of changes in trends (plus/minus/stable) within the differences between 2 consecutive values of symptom medication score.

### Relationship of Quality Index Values With Adherence to Recording

No significant differences were observed between the groups of highly and poorly adherent patients with regard to the average intravariation index, percentage of zero values, and the coefficient of variation. Of note, the coefficient of variation of the VAS score was higher among highly than poorly adherent patients, but the difference was only marginally significant (*P*=.06). Similarly, the average intravariation index of the RTSS was higher among patients with adherence to recording of 80% or more, but the difference did not reach statistical significance (*P*=.08). In contrast, the percentage of changes in trend was significantly lower among patients who recorded their symptoms on less than 80% of the prescribed days compared to highly adherent patients, considering each of the 3 indexes: RTSS (*P*<.001), VAS (*P*<.005), and CSMS (*P*<.001) (Table 3, Figure 3).

## Discussion

We retrospectively analyzed patient-generated data recorded during the grass pollen season by patients with seasonal allergic rhinitis to grasses. The collected data contained information on daily symptoms (RTSS), a combination of symptoms and medication intake (CSMS), and the overall impact of pollen allergy on daily life (VAS). Our analysis shows that (1) RTSS, CSMS, and VAS trajectories correlate well over time at population level; (2) VAS average values correlate well with average values of RTSS and CSMS at individual level; (3) the percentage of days with a change in trend during the observation period is higher in patients with high adherence to recording; and (4) other investigated parameters such as the percentage of zero values, the coefficient of variation, and the intravariation index are not significantly different among highly versus poorly adherent patients. Overall, our results suggest that the percentage of days with a change in trend deserves further investigation in a prospective study as a proxy of data quality in patients monitoring their pollen allergy with an electronic diary app.

Electronic diaries are increasingly produced and used in medicine, particularly in allergology. Nevertheless, studies focusing on the accuracy and completeness of the patient-generated information collected via electronic diaries are substantially missing. This is of high priority, as data validation is a prerequisite of any scientific or clinical use of the information collected through mobile apps from patients. A recent study demonstrated that daily monitoring with a VAS score has a high intrarater reliability and medium-high validity, reliability, and responsiveness, suggesting the validity of this simple methodology in monitoring disease impact on the patient’s daily life [32]. Along the same line of evidence, our study demonstrates that VAS correlates well with complex measurements such as RTSS and CSMS, both in terms of trajectory at the population level (Figure 1) and as average values at the individual level (Figure 2).

The percentage of days with changes in trend within the registration period is an interesting parameter, as it can be examined within the context of any trajectory, independently from the structure of the algorithm generating the clinical score. Therefore, this parameter can be applied to VAS, RTSS, CSMS, or any other index that will be generated and validated in the future. We speculate that patients whose personal interest in the recording of their electronic diary is lower, may still be adherent but inaccurate, replicating the same pattern of values every day. The day-to-day variability of pollen counts coupled with daily variability of exposure to pollen as well as the use of preventative medication may impact markedly on day-to-day variability in symptom score. A patient highly adherent to recording is better placed to record the symptom variability and thus more likely to report a higher number of changes in trend. With regard to the use of electronic diaries in clinical practice and research, a tool to predict the quantity and quality of expected data collection would be helpful. Unfortunately, to our knowledge, no such tools exist at the moment. In a previous approach to assess and predict the adherence of patients to symptom recording of patients with pollen allergies, we observed an association between the reporting behavior between the 7th and 21st day of recording compared to the rest of the monitoring period (up to 70 days) [27].

The lack of a previously established methodology also justifies our explorative approach in the attempt to identify new statistical methods or methods generated in other contexts to address a novel research question. We therefore speculate that future studies will adopt similar approaches and generate new and even more precise methodologies to answer the same research questions. Further, an expansion to other chronic diseases for which digital data collection has been well adopted, for example, asthma, will be of great value.

We are aware of the limitations of our analysis. First of all, we retrospectively examined in an opportunistic approach a database already generated with different targets. Consequently, our paper can only generate the hypothesis that the percentage of changes in trend is a valuable parameter measuring the quality of patient-recorded data. Unfortunately, other important parameters such as clinical validity could not be investigated within this data set, as this parameter should be investigated independently from the adherence to reporting. Therefore, before any use of changes in trend as a parameter in clinical practice, our hypothesis must be prospectively proven in studies designed with this specific scope. Second, we limited our investigation period to a maximum of 56 days of recording and in the context of a clinical investigation. The generalizability of our conclusions to a real-life setting and to longer periods of monitoring are also to be proven in real-world studies and longer observation periods. Third, our study population was composed of children, whose electronic diary recording is partially (in general until the age of 14 years) performed with the assistance of parents and whose influence on the reliability of data should also be accounted for. Fourth, we have used adherence to recording as a reference parameter under the assumption that patients more compliant in regularly filling their electronic diaries are also those whose data are more reliable. This assumption also should be proven in a new prospective study by adopting external quality standards not affected by recording patterns. Fifth, there is no possibility of correlating medication usage with adherence to digital symptom recording.

In conclusion, our retrospective analysis identifies the percentage of changes in trend in the trajectory of RTSS, CSMS, and VAS as a parameter, intrinsic to the trajectory itself, thereby representing a valuable candidate as proxy measure of data quality. This hypothesis deserves now to be investigated in prospective studies.

## Appendix

Multimedia Appendix 1 Supplementary data.

## Abbreviations

CSMS: Combined Symptom and Medication Score
RTSS: Rhinoconjunctivitis Total Symptom Score
VAS: Visual Analogue Scale
