# Supplementary material for: Validation Parameters of Patient-Generated Data for Digitally Recorded Allergic Rhinitis Symptom and Medication Scores in the @IT.2020 Project: Exploratory Study
Source: JMIR Mhealth Uhealth. 2022 Jun 3;10(6):e31491. doi: 10.2196/31491 (PMC9206201; doi:10.2196/31491)
Supplement: Multimedia Appendix 1 [file mhealth_v10i6e31491_app1.docx]

**Multimedia Appendix 1**

**Figure S1 –** Gramineae pollen counts (g/m^3^) detected between March and September 2016 in Rome;

Whole and peak season periods were highlighted according to locally adapted EAACI (European Academy of Allergy and Clinical Immunology) criteria in dark and light green, respectively. The exact dates for grass pollination periods were: **Whole SEASON**: 13/04-18/07; **Peak SEASON**: 04/05-28/06; **High days:** 23/04, 30/04, 04-05/05, 07-11/05, 13/05, 16-23/05, 26-27/05, 29/05, 31/05-02/06, 04-06/06, 08-09/06, 11-19/06, 22/06, 24/06, 26-27/06, 02/07, 10/07, 15/07.

**Figure S2** – Daily adherence to recording for all 101 patients during the peak grass pollen season. Green: days with completed questionnaire within the prescribed monitoring period; red: missed reporting days; blue: days of delayed reporting start or early reporting end within the prescribed monitoring period.

**Figure S1**

**Figure S2**

| **Table S1.** Atopic sensitization to airborne allergens (SPT ≥3 mm) | | | | | | | | |
| --- | --- | --- | --- | --- | --- | --- | --- | --- |
|  |  |  |  |  |  |  |  |  |
|  |  |  |  |  |  |  |  |  |
|  |  |  |  | **n=101** | |  |  |  |
|  |  |  |  | **n** | **%** |  |  |  |
|  | ***Outdoor allergens*** | |  |  |  |  |  |  |
|  |  | Timothy grass |  | 98 | *97.0* |  |  |  |
|  |  | Bermuda grass |  | 91 | *90.1* |  |  |  |
|  |  | Olive tree |  | 89 | *88.1* |  |  |  |
|  |  | Cypress |  | 83 | *82.2* |  |  |  |
|  |  | Birch |  | 47 | *46.5* |  |  |  |
|  |  | Hazel |  | 47 | *46.5* |  |  |  |
|  |  | Wall pellitory |  | 68 | *67.3* |  |  |  |
|  |  | Plane tree |  | 60 | *59.4* |  |  |  |
|  |  | Alternaria |  | 48 | *47.5* |  |  |  |
|  |  | Russian thistle |  | 46 | *45.5* |  |  |  |
|  |  | Mugwort |  | 36 | *35.6* |  |  |  |
|  |  | Ragweed |  | 38 | *37.6* |  |  |  |
|  |  |  |  |  |  |  |  |  |
|  | ***Indoor allergens*** | |  |  |  |  |  |  |
|  |  | House Dust Mite |  | 74 | *73.3* |  |  |  |
|  |  | Dog dander |  | 80 | *79.2* |  |  |  |
|  |  | Cat dander |  | 71 | *70.3* |  |  |  |
|  |  |  |  |  |  |  |  |  |
